# Three-Dimensional Map of Isoprognostic Zones in Glottic Cancer Treated by Transoral Laser Microsurgery as a Unimodal Treatment Strategy

**DOI:** 10.3389/fonc.2018.00175

**Published:** 2018-05-22

**Authors:** Cesare Piazza, Marta Filauro, Alberto Paderno, Filippo Marchi, Pietro Perotti, Riccardo Morello, Stefano Taboni, Giampiero Parrinello, Fabiola Incandela, Andrea Iandelli, Francesco Missale, Giorgio Peretti

**Affiliations:** ^1^Department of Otorhinolaryngology – Head and Neck Surgery, Fondazione IRCCS, National Cancer Institute of Milan, University of Milan, Milan, Italy; ^2^Department of Otorhinolaryngology – Head and Neck Surgery, University of Genoa, Genoa, Italy; ^3^Department of Otorhinolaryngology – Head and Neck Surgery, University of Brescia, Brescia, Italy

**Keywords:** transoral laser microsurgery, laryngeal cancer, glottic cancer, carbon dioxide laser, prognosis, oncologic outcomes

## Abstract

**Introduction:**

The Union for International Cancer Control–American Joint Committee on Cancer TNM staging system for glottic squamous cell carcinoma (SCC) includes different types of lesions defined by the involvement of specific subsites in each T category. Our study aims to identify different subcategories according to tumor local extension and determine oncologic outcomes after treatment by transoral laser microsurgery (TLM) alone.

**Methods:**

We retrospectively evaluated 410 patients affected by previously untreated pT1-pT3 glottic SCC treated by TLM alone from January 2005 to December 2015 at the Departments of Otorhinolaryngology—Head and Neck Surgery, Universities of Genoa and Brescia, Italy. All patients had at least 2 years of follow-up. Clinical, radiological, surgical, and histopathological data were reviewed and tumors divided into six subcategories: I, pT1a not involving the anterior commissure (AC); II, pT1b involving the AC; III, pT2 extending superficially to the supraglottis or the subglottis; IV, pT2 infiltrating the vocal muscle; V, pT3 involving the anterior paraglottic space; VI, pT2 or pT3 with vertical extension across the AC with/without involvement of the pre-epiglottic space. Recurrence-free survival (RFS), local control with laser alone (LCL), and organ preservation (OP) were defined as the primary oncologic outcomes.

**Results:**

The 2, 5, and 10-year RFS for the entire series were 85.7, 80.3, and 73.8%, LCL rates 93.8, 92.1, and 89.6%, and OP rates 96.8, 95.9, and 93.5%, respectively. However, when comparing the rates of RFS, LCL, and OP for each subcategory, important differences emerged. In particular, subcategories V and VI showed a significantly increased risk of local recurrence [hazard ratio (HR) = 9.2 and 13.3, respectively]. These subcategories also had a significantly reduced probability to achieve LCL (HR: 73.6 and 93.5, respectively) and OP (HR: 6.4 and 8.1, respectively).

**Conclusion:**

The present classification in subcategories allows introducing the concept of a three-dimensional map of isoprognostic zones in glottic SCC treated by TLM alone as a useful tool in its management by a multidisciplinary tumor board.

## Introduction

Modern literature is replete of clear-cut evidence in favor of the good oncologic and functional outcomes of transoral laser microsurgery (TLM) for treatment of T1–T2 and selected T3 glottic squamous cell carcinomas (SCC) (1–14). Accurate and tailored patient selection, application of a multi-step diagnostic work-up based on pre- and intraoperative endoscopy, surgically oriented imaging assessment by dedicated head and neck radiologists, use of a state-of-the-art laser technology, and knowledge of pathways of spreading and recurrence have allowed clinicians to obtain, in large-volume oncologic centers, outstanding and reproducible results. However, even the 8^th^ Edition of the TNM staging system (15) is definitively too simplistic to precisely define the different possible extensions of glottic tumors (especially when dealing with T2 and T3 lesions), thus limiting a proper patient and interdisciplinary counseling. In fact, these T categories may differ according to the involvement of various glottic subsites, superficial spreading to the supraglottis or subglottis, deep extension toward visceral compartments [paraglottic (PGS) and pre-epiglottic spaces (PES)], vocal cord/arytenoid mobility, and infiltration of the cartilaginous framework.

Too frequently, even in well-structured multidisciplinary boards, the “T–N categories criterion” is the only one used to define a lesion and, therefore, to choose among the most appropriate therapeutic strategies. Indeed, our experience (4, 5, 9), as well as those of others (6–8), clearly shows that profound differences exist among tumors usually grouped together under the same T category but, in reality, presenting very different surgical challenges, oncological prognosis, and possible functional sequelae. The process of subdividing each T category in a number of more homogeneous subgroup of lesions should help in communicating and sharing therapeutic outcomes to be achieved in every single case scenario, thus making a custom tailored approach for evaluation and treatment of a specific tumor more feasible.

The present study introduces a new conceptual tool that we have defined “three-dimensional (3D) map of isoprognostic zones,” which allows for the definition, for each therapeutic strategy, of the oncologic outcomes that are reasonably obtainable for each specific T subcategory, more precisely than previously obtained by the simple use of TNM stratification alone. Herein, we embraced such a subcategorization to depict an isoprognostic zones map related to the use of TLM as a unimodal treatment strategy for management of early-intermediate glottic SCCs. However, the same process could be extended to other T and N categories, laryngeal subsites, or head and neck sites, as well as to other surgical and non-surgical treatment modalities, taken alone or in various combinations.

The aim of the present study is to retrospectively stratify in 6 subcategories, defining an equal number of isoprognostic zones, a cohort of 410 T1, T2, and selected T3 glottic SCCs according to location and extension, describe different patterns of growth and possible pathways of recurrence, and define the role and limits of TLM as a single treatment modality in terms of recurrence-free survival (RFS), local control with laser alone (LCL), and organ preservation (OP) rates for each subcategory.

## Materials and Methods

We retrospectively evaluated 410 patients (371 males and 39 females) affected by previously untreated T1–T3 glottic SCC managed by TLM alone from January 2005 to December 2015 at the Departments of Otorhinolaryngology—Head and Neck Surgery, Universities of Genoa and Brescia, Italy. Patients age ranged from 31 to 94 years (median, 68), and all had at least 2 years of follow-up (range, 24–120 months; median, 72). Inclusion criteria were glottic pT1, pT2, and selected pT3 SCC with evidence of limited anterior PGS and/or PES involvement and adequate laryngeal exposure preoperatively assessed by the Laryngoscore (16). Patients affected by T3 glottic SCC with minimal laryngeal framework invasion or arytenoid fixation due to posterior PGS involvement and those previously treated elsewhere with TLM, radiotherapy (RT), chemoradiotherapy (CRT), or open partial horizontal laryngectomies (OPHL) were excluded from the present study.

Preoperative flexible videolaryngoscopy assessed superficial tumor margins under white light (WL) and narrow band imaging (NBI, Olympus Medical System Corporation, Tokyo, Japan). Deep neoplastic extension was evaluated using contrast enhanced imaging [computed tomography or magnetic resonance (MR)] performed by dedicated head and neck radiologists. Neck ultrasound with or without fine needle aspiration cytology was routinely performed in lesions with significant supraglottic extension or those defined as glottic cT3. Intraoperative rigid endoscopy with 0°, 30°, and 70° telescopes under WL and NBI was accomplished during microlaryngoscopy to obtain better definition of superficial surgical margins (17). All patients underwent TLM using previously described surgical techniques and instrumentation (9, 16). Patients with clinically negative neck nodes received purely local treatment by TLM. Four patients with clinically positive neck disease underwent unilateral selective (levels II–V) neck dissection. Transoral re-excision was accomplished in case of deep or more than one superficial positive margins. We also excluded patients who received adjuvant treatments (RT and/or CRT) due to the presence of persistent tumor after re-excision, perineural invasion, angioembolization, multiple positive lymph nodes, or extracapsular spread. We retrospectively reviewed clinical, radiological, surgical, and histopathological data, and divided tumors accordingly into six subcategories: I, pT1a not involving the anterior commissure (AC); II, pT1b involving the AC or both vocal folds; III, pT2 extending superficially to the supraglottis or the subglottis; IV, pT2 infiltrating the vocal muscle (VM); V, pT3 involving the anterior PGS; VI, pT2 or pT3 with trans-AC extension with or without involvement of the PES.

All tumors were restaged according to the eighth edition of the Union for International Cancer Control—American Joint Committee on Cancer TNM staging system (15). At the time of diagnosis, the cT categories were as follows: 284 cT1a-b, 84 cT2, and 42 cT3. The pT stage and the ensuing division in subcategories were 211 pT1a (subcategory I); 61 pT1b (subcategory II); 45 pT2 (subcategory III); 41 pT2 (subcategory IV); 40 pT3 (subcategory V); and 12 pT2-pT3 (subcategory VI).

No approval from the Ethics Committee for this study was deemed necessary at our Institutions after a formal request to the appropriate parties. Each patient signed an informed consent for treatment of personal data for scientific purposes before enrollment.

### Statistical Analysis

Statistical analysis was carried out using STATA 13 software (StataCorp. 2013. Stata Statistical Software: Release 13. College Station, TX, USA: StataCorp LP). 2-, 5-, and 10-year RFS, LCL, and OP rates were evaluated by the Kaplan–Meier product limit estimate. Subcategories were first individually compared among each other by the log-rang test (I vs. II, II vs. III, III vs. IV, IV vs. V, and V vs. VI) to identify major differences in terms of RFS, LCL, and OP. Subcategory I has been considered the reference point [hazard ratio (HR) = 1] when evaluating RFS and LCL. Conversely, the reference point for OP was subcategory II, due to the lack of events (total laryngectomies) in subcategory I. Furthermore, a Cox proportional hazard regression model was employed to quantify the stratification potential of subcategories (considered as a categorical variable) and to obtain the related HR for RFS, LCL, and OP. No further variable was included in the regression model, since a different distribution of risk factors was not considered as a confounding element, but as one of the bases for the subcategory stratification itself. Comparisons between survival curves were carried out using the log-rank test. The final time-point for RFS was the date of the first recurrence (patients who died without any recurrence were considered as censored). The final time-point for LCL was the date of any further open or non-surgical treatment for relapse. The final time-point for OP was the date of total laryngectomy (patients who died without a previous total laryngectomy were considered as censored).

## Results

Fifty-nine (14.4%) patients had recurrences. Forty-nine had a local recurrence: 13 in subcategory I (6% of patients in such subcategory), 11 in subcategory II (18%), 7 in subcategory III (15%), 6 in subcategory IV (15%), 8 in subcategory V (20%), and 4 in subcategory VI (33%). Nine patients experienced loco-regional recurrence: 1 in subcategory III (2%), 3 in subcategory IV (7%), 3 in subcategory V (8%), and 2 in subcategory VI (17%). Only one patient in subcategory V (2%) had an exclusively regional recurrence. Salvage treatment was represented by TLM in 40 patients, OPHL with uni- or bilateral selective neck dissection in 6, total laryngectomy with uni- or bilateral neck dissection in 13 (4 of them followed by CRT), RT alone in 1, and chemotherapy alone in the only patient with neck failure. One patient received best supportive care due to refusal of any further treatment. At the last follow-up (December 2017), 57 patients had died: 8 for disease progression, and 49 for other causes. Four patients were alive with disease, while in the remaining 349 there was no evidence of disease.

**Figure 1:**
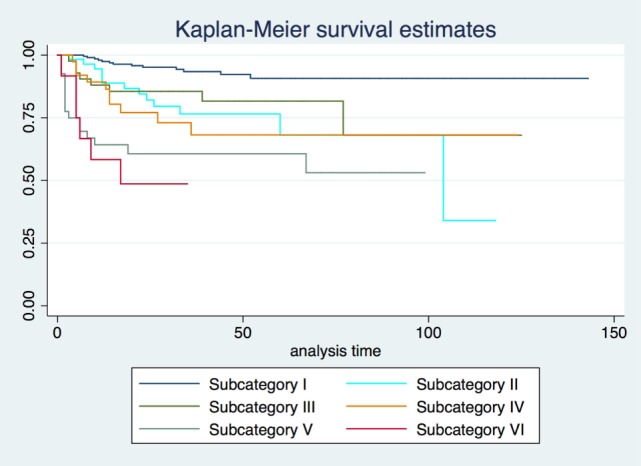
Recurrence-free survival Kaplan–Meier curves for each subcategory.

**Figure 2:**
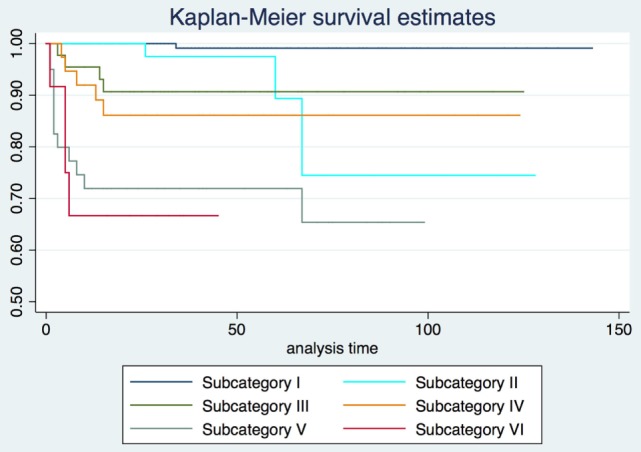
Local control with laser alone Kaplan–Meier curves for each subcategory.

**Figure 3:**
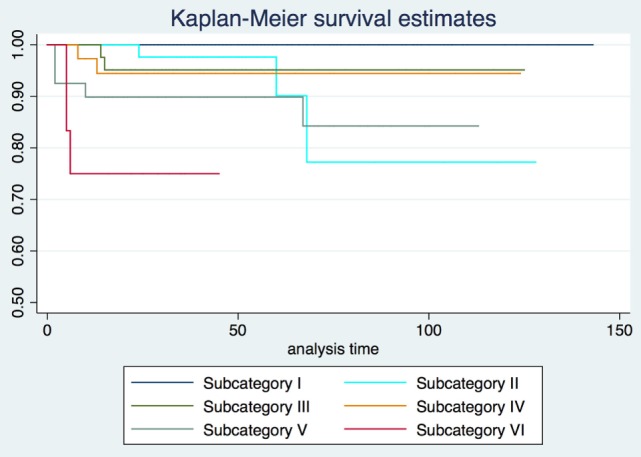
Organ preservation Kaplan–Meier curves for each subcategory.

2-, 5-, and 10-year RFS rates for the entire series were 85.7, 80.3, and 73.8%, LCL rates were 93.8, 92.1, and 89.6%, and OP rates were 96.8, 95.9, and 93.5%, respectively. RFS, LCL, and OP rates according to each pT subcategory are detailed in Table 1 and Figures 1–3. Progressively comparing the estimates of the hazard functions of each subcategory with the following one, we evidenced a significant difference at the log-rank test between subcategory I and II (*p* = 0.004), as well as IV and V (*p* = 0.050) when considering LCL. The significant difference between subcategory I and II was also confirmed when considering RFS and OP (both *p* < 0.001).

**Table 1:** Recurrence-free survival (RFS), local control with laser alone (LCL), and organ preservation (OP) at 2, 5, and 10 years for each subcategory.

	RFS			LCL			OP		
			
	2 years (%)	5 years (%)	10 years (%)	2 years (%)	5 years (%)	10 years (%)	2 years (%)	5 years (%)	10 years (%)
Overall series	85.7	80.3	73.8	93.8	92.1	89.6	96.8	95.9	93.5
Subcategory I	95.1	90.7	90.7	100	99.1	99.1	100	100	100
Subcategory II	82.1	68.0	–	100	89.4	74.5	97.6	90.1	77.2
Subcategory III	85.5	81.6	68.0	90.7	90.7	90.7	95.1	95.1	95.1
Subcategory IV	77.1	68.1	68.1	86.1	86.1	86.1	94.4	94.4	94.4
Subcategory V	60.7	60.7	–	71.9	71.9	–	89.9	89.9	–
Subcategory VI	48.6	–	–	66.7	–	–	75	–	–

In the Cox proportional hazards regression analysis, according to our patient stratification, subcategories V and VI showed an approximately 10-fold increase in the risk of local recurrence, with an hazard ratio (HR) of 9.2 and 13.3, respectively (*p* < 0.001). In addition, the same subcategories had a greatly reduced probability to achieve LCL (HR = 73.6 and 93.5, respectively; *p* < 0.001) and OP (HR = 6.4 and 8.1, respectively; *p* < 0.01). HRs concerning RFS, LCL, and OP for each subcategory are detailed in Table 2.

**Table 2:** Cox analysis between subcategories of recurrence-free survival (RFS), local control with laser alone (LCL), and organ preservation (OP).

	RFS			LCL			OP		
			
	Hazard ratio (HR)	95% CI	*p*-Value	HR	95% CI	*p*-Value	HR	95% CI	*p*-Value
Subcategory I	1.0	–	–	1.0	–	–	–	–	–
Subcategory II	4.0	1.86–8.68	<0.001	11.4	1.18–109.40	0.035	1.0	–	–
Subcategory III	3.1	1.28–7.50	0.012	18.8	2.11–168.50	0.009	1.7	0.37–7.41	0.508
Subcategory IV	4.5	1.98–10.33	<0.001	28.2	3.29–241.09	0.002	2.5	0.59–10.31	0.218
Subcategory V	9.2	4.45–19.33	<0.001	73.6	9.55–566.86	<0.001	6.4	1.80–22.91	0.004
Subcategory VI	13.3	5.01–35.27	<0.001	93.5	10.41–840.35	<0.001	8.1	1.80–36.18	0.006

**Figure 4:**
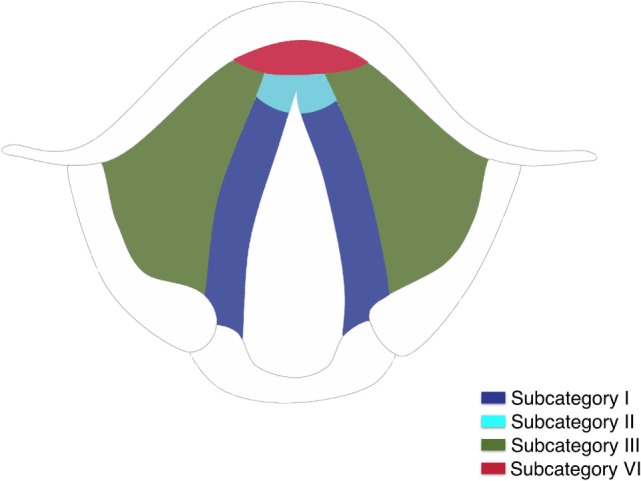
Three-dimensional map of the isoprognostic zones I, II, III, and VI, view from above.

**Figure 5:**
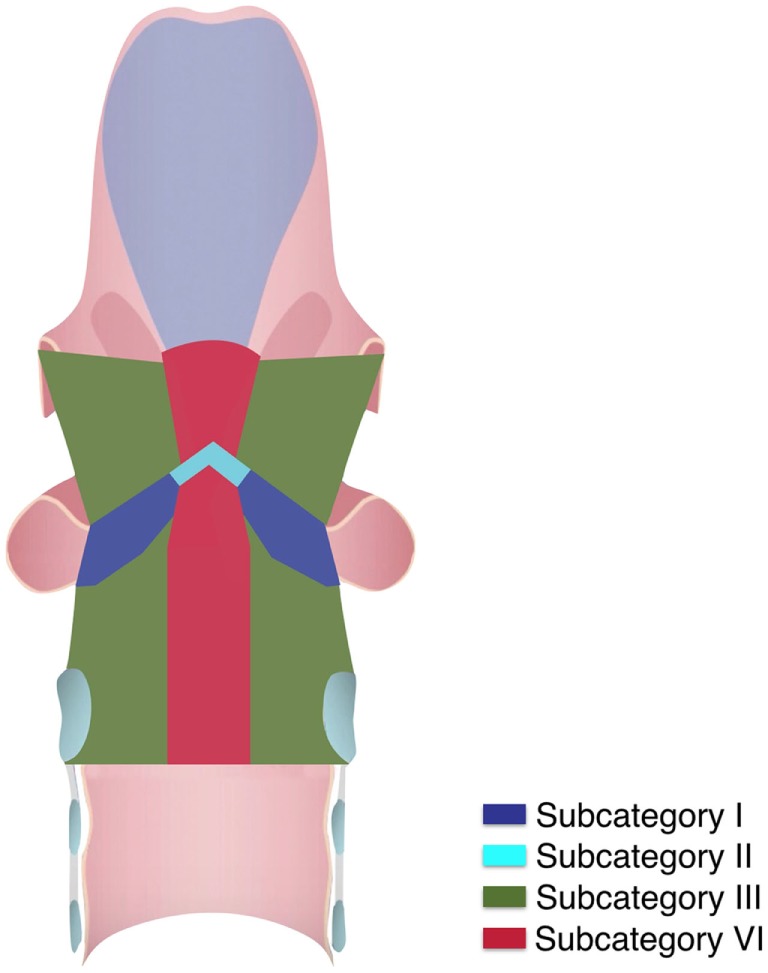
Three-dimensional map of the isoprognostic zones I, II, III, and VI, view from inside.

**Figure 6:**
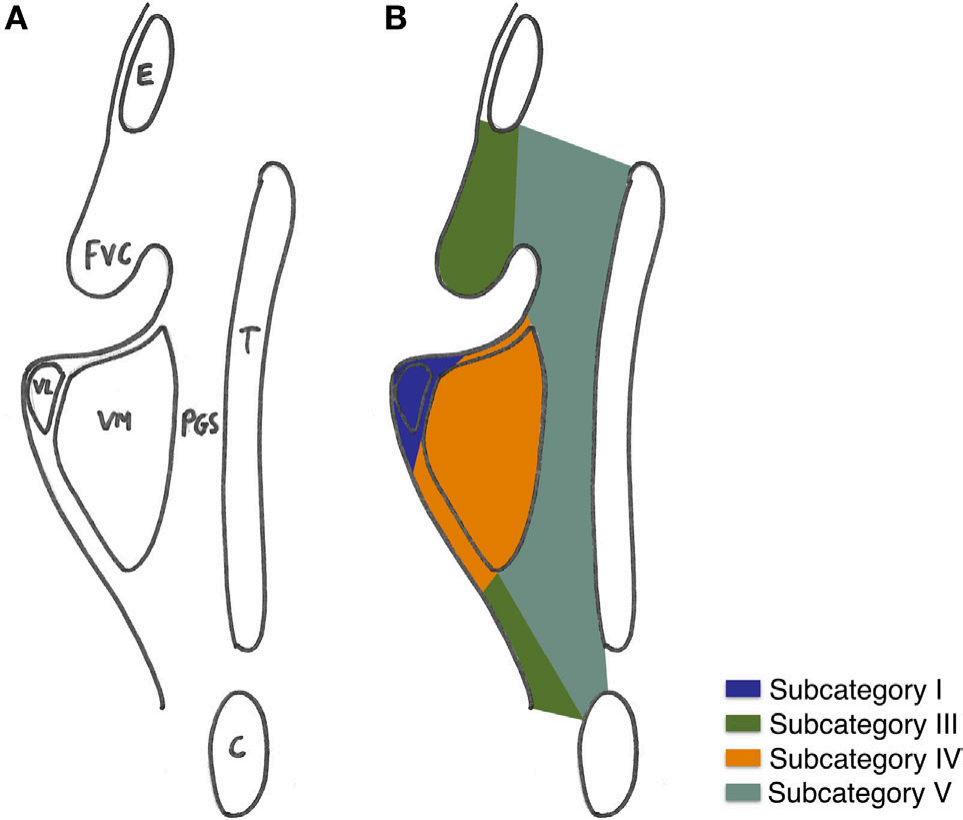
**(A)** Coronal view of the right hemilarynx, anterior half (E, epiglottis; FVC, false vocal cord; VL, vocal ligament; VM, vocal muscle; PGS, anterior paraglottic space; T, thyroid cartilage; C, cricoid) and **(B)** three-dimensional map of the isoprognostic zones I, III, IV, and V, coronal view of the right hemilarynx, anterior half.

Three-dimensional maps of the isoprognostic zones for glottic SCCs treated by TLM alone are depicted in Figures 4–6.

## Discussion

The introduction of a novel conceptual tool such as the present that we defined “3D map of isoprognostic zones” tries to overcome, in our opinion, one of the most limiting factors in the contemporary literature concerning TLM for laryngeal SCC. In fact, oncologic outcomes of such a technique (as well as those of many other surgical and non-surgical therapeutic options) are frequently reported on the simple basis of T category, if not stage, placing a number of different lesions such as T1a, T1b, T2, and limited T3 under the same umbrella term of “early-intermediate” tumors. The ensuing overall outcomes may, therefore, seem quite encouraging even though, in reality, a much more differentiated scenario can be described when one specifically peruses the T category and, especially, applies more detailed subcategorization, as herein reported, that is able to consider specific issues. Such an approach directly derives from the daily use of an intraoperative microscopic view of such lesions, coupled with systematic application of modern biologic endoscopy techniques, and high-resolution radiologic imaging. All these tools are more articulated and time-consuming but, nonetheless, provide a comprehensive description of the different possible pathways of spread of glottic SCCs. In fact, superficial as well as deep progression of the lesion may harbor different challenges in terms of local control and OP. In particular, the present data confirm those already published by our group (5, 9, 18, 19) and together form the basis of the conceptualization attempted by the 3D map of isoprognostic zones herein described.

Our results confirm both the outstanding outcomes for the entire cohort in terms of RFS, LCL, and OP rates obtained by TLM alone and the need to analyze each subcategory to better highlight the real value of a treatment in each isoprognostic zone. Indeed, the present survival curves are effectively stratified according to our proposal of subcategorization. The HR for RFS was 4.5, 9.2, and 13.3 times significantly higher in subcategories IV, V, and VI, respectively, thus emphasizing that the vertical extension across the AC with/without involvement of the PES should be considered as a real weakness for the TLM approach. Such a crucial issue is also confirmed by the reduced LCL and the higher need for salvage total laryngectomy in subcategory VI compared with other isoprognostic zones of the 3D map.

In our opinion, subcategory VI has the worst outcome in the present series since transglottic SCCs tend to spread from the PES upwards into the supraglottis and hence outside the larynx due to the absence of a significant anatomical barrier in this area, or directly through the crico-thyroid membrane at the subglottic level. Moreover, the tangential transoral microscopic visualization of the supra- and subcommissural areas may further penalize adequate surgical treatment of tumors deeply involving these visceral compartments.

When sequentially comparing the single subcategories, it is possible to observe a significant shift in LCL from subcategory I to II–III, as well as from IV to V–VI. This may be seen as the passage from a superficially confined neoplasm (subcategory I) to a superficially spreading tumor (subcategories II–III), finally shifting to a deeply infiltrating lesion involving the laryngeal visceral spaces (subcategories V–VI).

Still, our results, in line with the literature, highlight how patients in the IV subcategory have a worse outcome compared to the III subcategory, even if both belong to the pT2 category, while those included in subcategory V for deep extension to the anterior PGS (limited pT3 lying in front of a plane tangential to the arytenoid vocal process and perpendicular to the ipsilateral thyroid lamina) have comparable outcomes with other traditional therapeutic options (20–22).

In light of this, subcategories IV, V, and VI represent the highest-risk group of patients to be managed by TLM alone. Thus, as already demonstrated in previous reports (23–25), they merit planned radiological examination (better with MR) even in the absence of clinical and endoscopic doubts during follow-up, due to their well-known propensity to recur submucosally.

In conclusion, we can assume that the propensity to use the term “early-intermediate stage” to describe a wide spectrum of diseases ranging from lesions limited to one vocal cord to those with a trans-AC extension as well VM infiltration or invasion of the anterior PGS and/or PES, may risk erroneous inclusion in a given prognostic category, with subsequent application of suboptimal treatment strategies (26–28). Ideal oncological and functional outcomes require accurate and comprehensive pre- and intraoperative diagnostic work-up, specialized skills, and expertise. Poor judgment will increase the risk of recurrence or the need for multimodal treatments (with associated increased toxicity and sequelae) to achieve disease control and functional preservation.

We must not forget, however, that the isoprognostic zones presented herein are strictly related and influenced by the specific therapeutic approach chosen, i.e., in the present series, by TLM. However, this should not limit their application to other treatment modalities and, even more interestingly, could fuel a stimulating comparison between them, especially in the context of a multidisciplinary tumor board. Our ideal target in the future would be, therefore, to be able to precisely choose, for each lesion in a given patient, the most adequate therapeutic approach, in order to ensure the highest oncological success with the lowest impact in terms of morbidity and costs.

## Ethics Statement

No approval from the Ethics Committee for this study was deemed necessary at our Institutions after a formal request to the appropriate parties. Each patient signed an informed consent for treatment of personal data for scientific purposes before enrollment.

## Author Contributions

CP and GP: study design, conceptualization, definitive manuscript draft, and revision; MF, AP, FM, RM, ST, GiPa, FI, AI and FrMi: charts review, data collection, follow-up, and initial manuscript draft; AP and FrMi: statistical analysis; CP: drawings.

## Conflict of Interest Statement

The authors declare that the research was conducted in the absence of any commercial or financial relationships that could be construed as a potential conflict of interest.

## Abbreviations

3D, three-dimensional; UICC, Union for International Cancer Control; AJCC, American Joint Committee on Cancer; SCC, squamous cell carcinoma; TLM, transoral laser microsurgery; AC, anterior commissure; RFS, recurrence-free survival; LCL, local control with laser alone; OP, organ preservation; HR, hazard ratio; PGS, paraglottic space; PES, pre-epiglottic space; RT, radiotherapy; CRT, chemoradiotherapy; OPHL, open partial horizontal laryngectomy; WL, white light; NBI, narrow band imaging; CT, computed tomography; MR, magnetic resonance.
